# Compression hemostasis using fully covered self-expandable metallic stents for refractory hemorrhages caused by esophageal cancer: A pilot study

**DOI:** 10.3389/fgstr.2023.1120795

**Published:** 2023-02-21

**Authors:** Yonghua Bi, Jianzhuang Ren, Xinwei Han

**Affiliations:** Department of Interventional Radiology, the First Affiliated Hospital of Zhengzhou University, Zhengzhou, China

**Keywords:** esophageal cancer, esophageal hemorrhage, self-expanding metal stent, compression hemostasis, complications

## Abstract

**Objective:**

Fully covered self-expandable metallic stents (SEMSs) have been widely used as a salvage therapy for patients with esophageal variceal bleeding. However, the role of fully covered SEMSs in the management of hemorrhage caused by esophageal cancer has not yet been established. We aimed to investigate the safety and efficacy of fully covered SEMSs as a salvage therapy for esophageal cancer-related hemorrhage.

**Methods:**

From September 2019 to March 2022, 17 patients, who underwent the insertion of fully covered SEMS for malignant esophageal hemorrhages, were retrospectively analyzed. Chest computed tomography (CT) scans and esophagographies were performed routinely to determine the location and length of the tumor. A fully covered SEMS was implanted under fluoroscopy. Baseline demographics were retrospectively collected, that is those for sex, age, previous treatment, comorbidities, lesion type, and stent size.

**Results:**

A total of 20 metal stents were placed in 17 patients, with a technical success rate of 100% and a hemostasis success rate of 88.2%. Stent removal was performed in three patients because of complications. No perioperative deaths were related to stent placement or removal. Five main complications (29.4%) were found after stent insertion. Stent migration and restenosis were observed in two patients (11.8%). Except for two perioperative deaths and one patient lost to follow-up, all remaining 14 patients were successfully followed up. At the end of follow-up, two patients had survived without obvious symptoms, and a total of 12 patients were dead owing to tumor progression (*n *= 10), severe infection (*n* = 1), and cerebrovascular accident (*n* = 1). The median overall survival was 13.8 months.

**Conclusion:**

Insertion of a fully covered SEMS may be a safe and effective means of the salvage management of refractory esophageal cancer-related hemorrhage, and its use in this context may lead to the development of innovative methods for compression hemostasis. However, further study with a larger sample size and comparison with other forms of salvage therapy.

## Introduction

Approximately half of all patients with esophageal cancer are diagnosed in the middle and advanced stages of the disease, that is the stages in which tumors are not completely resectable (1); those patients often have a poor prognosis, with a low 5-year overall survival rate (2–4). Esophageal hemorrhage occurs in approximately 1%–5% of patients with advanced esophageal cancer, which is a big challenge (5). As a first-line treatment of tumor hemostasis, many kinds of endoscopic treatment modalities have been used, including the application of heater probes, local injections of adrenaline, argon plasma coagulation, and cryotherapy, and radiofrequency ablation (6). However, rebreeding occurs frequently despite the variety of endoscopic interventions (7).

Self-expandable metallic stent (SEMS) placement has been served as palliative treatment for advanced esophageal patients with esophageal fistula or stricture (8–10). Fully covered SEMSs have been widely used as a salvage therapy for patients with esophageal variceal bleeding (11, 12). However, only a few case reports and case series, such as those on duodenal tumors (13, 14) and esophageal cancer (6, 12, 15), report the use of fully covered SEMSs for malignant tumors in the upper gastrointestinal track to achieve hemostasis after the failure of conventional endoscopic interventions.

In addition, it should be noted that massive esophageal hemorrhage is also observed following SEMS insertion for patients with malignant esophageal stricture or fistula (16–18). Currently, the role of fully covered SEMS in the management of hemorrhage caused by esophageal cancer is not established. In this study, we aimed to investigate the safety and efficacy of fully covered SEMS as a salvage therapy for esophageal cancer-related hemorrhage.

## Materials and methods

### Study design

From September 2019 to March 2022, 17 patients, who underwent the placement of fully covered SEMS, for malignant esophageal hemorrhage, were retrospectively analyzed. Exclusion criteria were the presence of a malignant airway stricture without airway stent placement or proximal esophageal cancer (both of which are contraindications to the insertion of esophageal stents); and the patient’s inability to comply with follow-up procedures. Baseline demographics were retrospectively collected, including sex, age, previous treatment, comorbidities, lesion type, and stent size. Ethics approval was waived by the Institutional Review Board of University because of the study’s retrospective nature. Written informed consent was obtained from all patients before they underwent stenting procedures.

### Stent insertion

**Figure 1:**
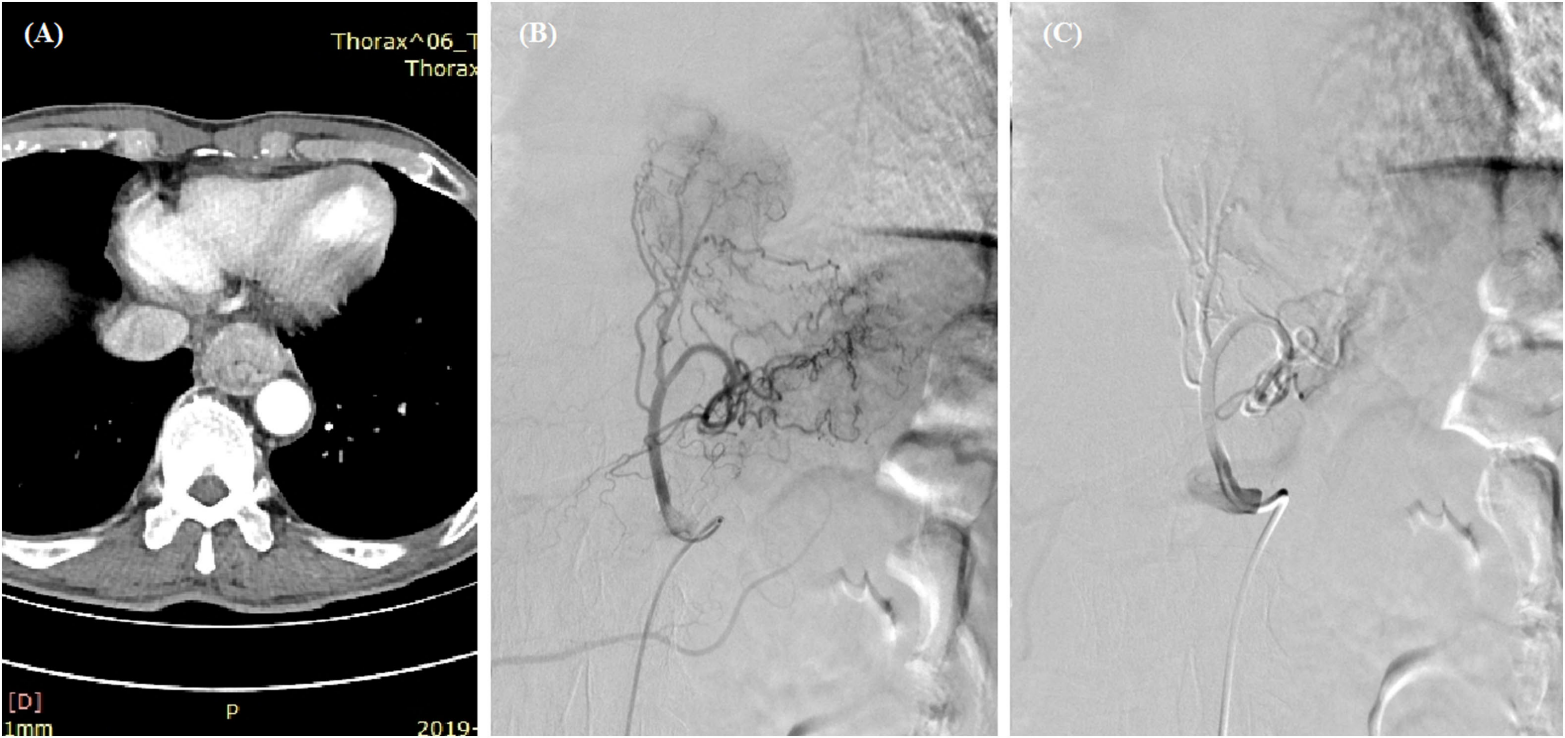
Transarterial chemoembolization for esophageal cancer. **(A)** A thickened esophageal wall was observed in the lower esophagus. **(B)** Staining of the tumor and its feeding artery were observed after angiography. **(C)** The left gastric artery was embolized with 350- to 560-μm polyvinyl alcohol particles after the infusion of 100 mg of oxaliplatin and 250 mg of fluorouracil.

**Figure 2:**
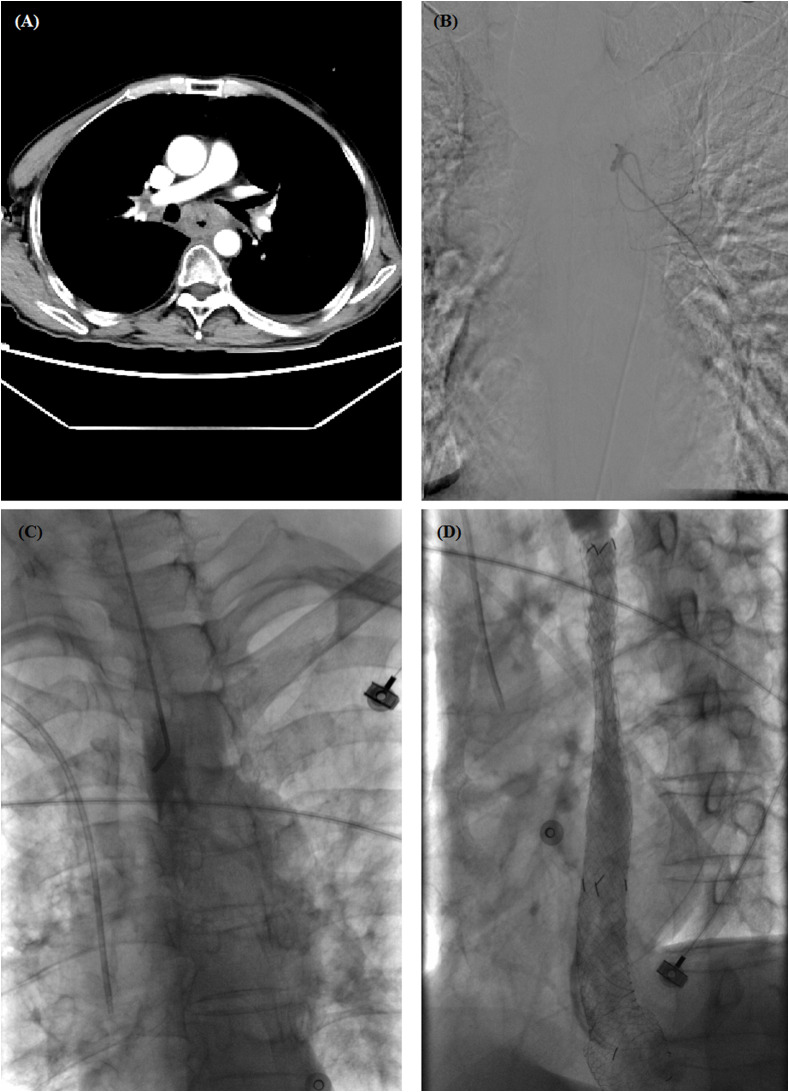
A 60-year-old male with esophageal hemorrhage treated by self-expandable metallic stent (SEMS) insertion. **(A)** Chest computed tomography (CT) scan showed a thickened wall in the middle esophagus. **(B)** Transarterial embolization was performed, but hemorrhage did not stop. **(C)** Esophagography showed complete obstruction in the middle esophagus. **(D)** Esophagography shows a good position and patency of stent after placement. CT, computed tomography; SEMS, self-expandable metallic stent.

**Figure 3:**
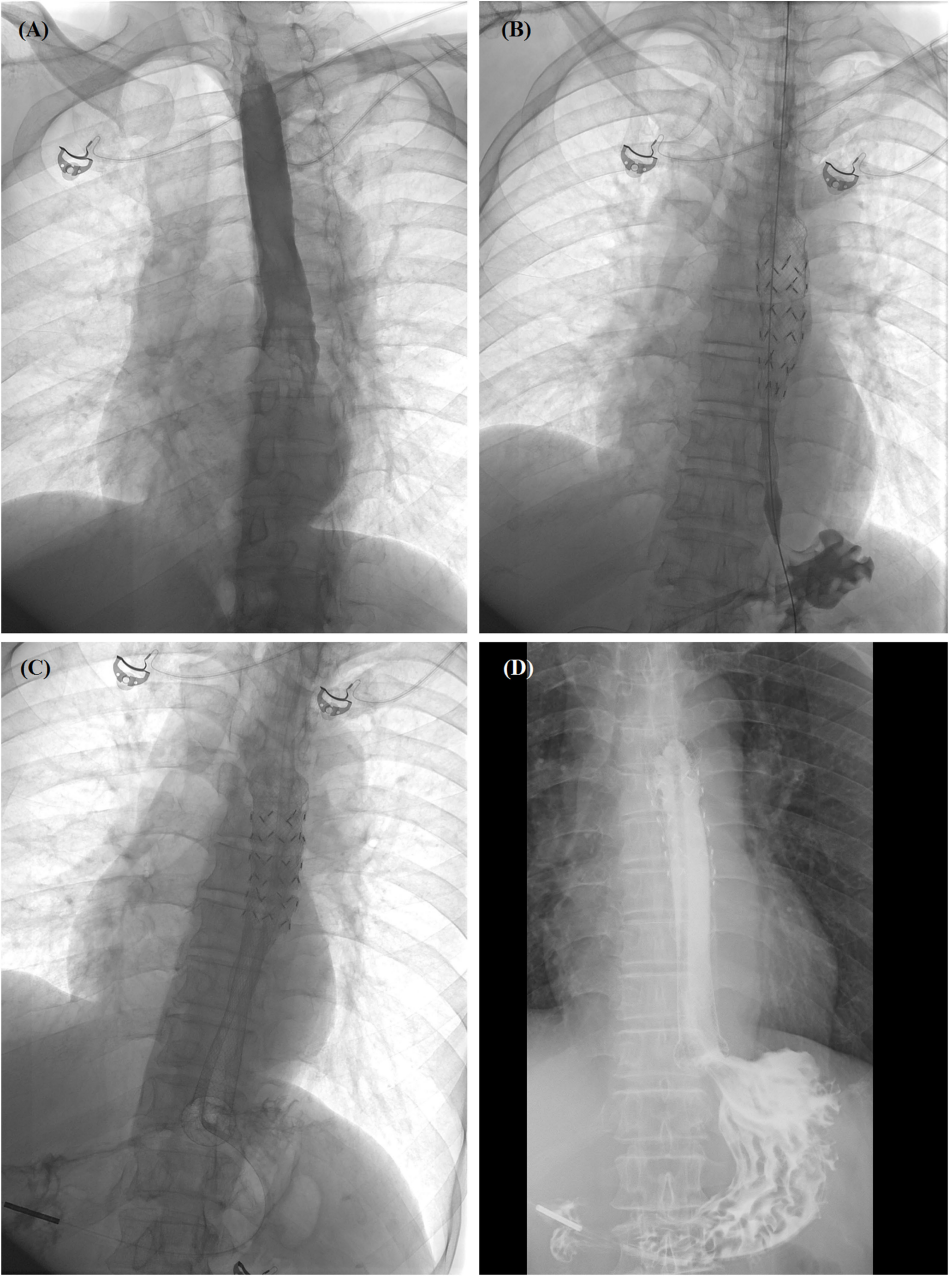
Self-expandable metallic stent (SEMS) insertion procedure. **(A)** Anteroposterior esophagography showed complete obstruction in the lower esophagus. **(B)** The delivery system of segmental radioactive metal stent was inserted, and stent migration happened. **(C)** Another fully covered SEMS was released by withdrawing the sheath. **(D)** Esophagography confirmed that the contrast agent passed smoothly after stent placement. SEMS, self-expandable metallic stent.

Chest computed tomography (CT) scans were performed before stenting to show the location and length of the esophageal cancer (**Figures 1A**, **2A**). The stent size was assessed according to the measured data, with a length of 2 cm longer than the tumor length. All stent placements and removal procedures were performed under local anesthesia and fluoroscopy. Esophagography was performed by using an oral iodine contrast agent to confirm the tumor site and length (**Figures 2C**, **3A**). A 5F catheter (Cook Corporation, Bloomington, IN, USA) was inserted and a fully covered SEMS was implanted along a stiff guide wire. Esophagography was performed immediately after stent placement to show the patency of the SEMS (**Figures 2D**, **3B, C**). A removal hook and 10- to 12-F-long sheaths were inserted for stent removal if necessary.

### Follow-up

All patients were followed up by telephone interview or outpatient visits until 9 July 2022, or until their death. During the follow-up period, chest CT scans and/or esophagoscopies were performed (**Figure 3D**). Cause of death and survival outcomes were recorded. The main complications identified were procedure-related death, massive hemorrhage, esophageal perforation, stent migration, or restenosis. The primary end point of this study was the hemostasis success rate. The median overall survival was calculated either from stent placement or diagnosis of the esophageal cancer. Patient safety and overall survival were the study’s secondary end points.

### Statistical analysis

Continuous variables were shown as means ± standard deviations (SDs) or medians with interquartile ranges (IQRs). The survival curve was calculated by Kaplan–Meier analysis (GraphPad; GraphPad Software, Inc, San Diego, CA, USA).

## Results

### Patient characteristics

A total of 17 patients (14 males and three females), were enrolled, with a mean age of 70.4 ± 10.7 years. Eight patients had esophageal squamous cell cancer, two patients had esophageal adenocarcinoma, and one patient had undifferentiated carcinoma. The remaining six patients were hospitalized in emergency cases for esophageal bleeding and had esophageal stents placed during these emergency visits, meaning that we could not obtain pathological results for them. Three (17.6%), seven (41.2%), and four patients (23.5%) had tumors in the upper, middle, and lower esophagus, respectively. One patient was diagnosed with recurrent cancer 4 years after surgical resection and the remaining 16 patients were diagnosed with advanced esophageal cancer. Eight patients received chemotherapy or radiotherapy before SEMS placement (**Table 1**). Two patients who underwent stent placement for coronary disease had additional antithrombotic therapy. On admission, 14 of the 17 patients enrolled had a normal international normalized ratio, a mean prothrombin time test time of 11.9 ± 1.4 s, and mean thrombocyte count of 106.6 ± 52.1. Before stent placement, the mean red cell count was (3.7 ± 0.4) × 10^9^/L, and the mean hemoglobin level was 110.2 ± 13.2 g/L.

**Table 1:** Patient characteristics at admission.

Parameters	Data
Patient number, *n*	17
Male, *n* (%)	14 (82.4%)
Mean age (years) ± SD	70.4 ± 10.7
Lesion types, *n* (%)	
Esophageal squamous cell cancer	8 (47.1%)
Esophageal adenocarcinoma	2 (11.8%)
Undifferentiated carcinoma	1 (5.9%)
Not available	6 (35.3%)
Recurrence after surgery, *n* (%)	1 (5.9%)
Previous systemic treatments, *n* (%)	12 (70.6%)
Chemotherapy	8 (47.1%)
Radiotherapy	8 (47.1%)
Immunotherapy	3 (17.6%)
Targeted therapy	1 (5.9%)
Comorbidities, *n* (%)	
Hypertension	5 (29.4%)
Coronary disease	3 (17.6%)
Old cerebral infarction	1 (5.9%)
Diabetes mellitus	1 (5.9%)
Mean red cell count (× 10^9^/L) ± SD	3.7 ± 0.4
Mean hemoglobin level (g/L) ± SD	110.2 ± 13.2
Location of tumor, *n* (%)	
Upper esophagus	3 (17.6%)
Middle esophagus	7 (41.2%)
Middle and upper esophagus	2 (11.8%)
Middle and lower esophagus	1 (5.9%)
Lower esophagus	4 (23.5%)

IQR, interquartile range; SD, standard deviation.

### Stent placement and removal

A total of 20 metal stents were placed, comprising two segmental radioactive metal stents, 17 Bonastents, and one Ultraflex stent. The median diameter of the stents was 20 mm (IQR 18–20 mm) and median length was 100 mm (IQR 100–120 mm). The median inpatient duration was 8.5 days, and the total hospitalization cost was 4.8 × 10^4^ renminbi. Stent placement was technically successful in all patients (100%). Two patients died of non-procedure-related esophageal hemorrhage after stent placement, with a hemostasis success rate of 88.2%. Hemostasis success was found in all three female patients and in 12 of the 14 male patients, indicating that there was no significant difference in hemostasis success rate between male and female patients. Stent removal was performed in one and two patients because of intolerance of stent (*n* = 1) and restenosis (*n* = 2), respectively. One patient had stent replacement 6.6 months later due to an esophageal fistula caused by tumor progression, and a new fully covered SEMS was placed. The median indwelling duration of all stents was 3.1 months (IQR 1.5–5.5 months). In addition, two patients received transarterial embolization and three patients received transarterial chemoembolization before stent placement (**Figures 1B, C**, **2B**).

### Complications

No perioperative deaths were related to stent placement or removal, and the stent placement process did not induce or aggravate hematemesis. One patient complained of discomfort after stent insertion. Five main complications (29.4%) were identified during and after stent placement. Stent migration was observed in two patients (11.8%); one patient needed additional stent placement, whereas the other patient, with mild migration, did not require management. Stent restenosis was observed in two patients after 4.2 months and 7.0 months, respectively. Stent removal was performed for two patients with stent restenosis (**Table 2**).

**Table 2:** Clinical data on stenting.

Variables	Data
Patient number, *n*	17
Inpatient duration (days), median (IQR)	8.5 (5.3–15.5)
Mean total hospitalization cost ± SD	4.8 ± 2.1
Indwelling duration of stent (months), median (IQR)	3.1 (1.5–5.5)
Technique success rate	100%
Hemostasis success rate	88.2%
Size of esophageal stents	
Stent length (mm), median (IQR)	100 (100–120)
Stent diameter (mm), median (IQR)	20 (18–20)
Complications, *n* (%)	5 (29.4%)
Stent migration	2 (11.8%)
Stent intolerance	1 (5.9%)
Stent restenosis	2 (11.8%)
Clinical outcomes, *n* (%)	
Lost to follow-up	1 (5.9%)
Survival	2 (11.8%)
Death owing to tumor progression	10 (58.8%)
Death owing to massive bleeding	2 (11.8%)
Non-tumor-related death	2 (11.8%)
Median survival (months)	13.8

IQR, interquartile range; SD, standard deviation.

### Follow-up

**Figure 4:**
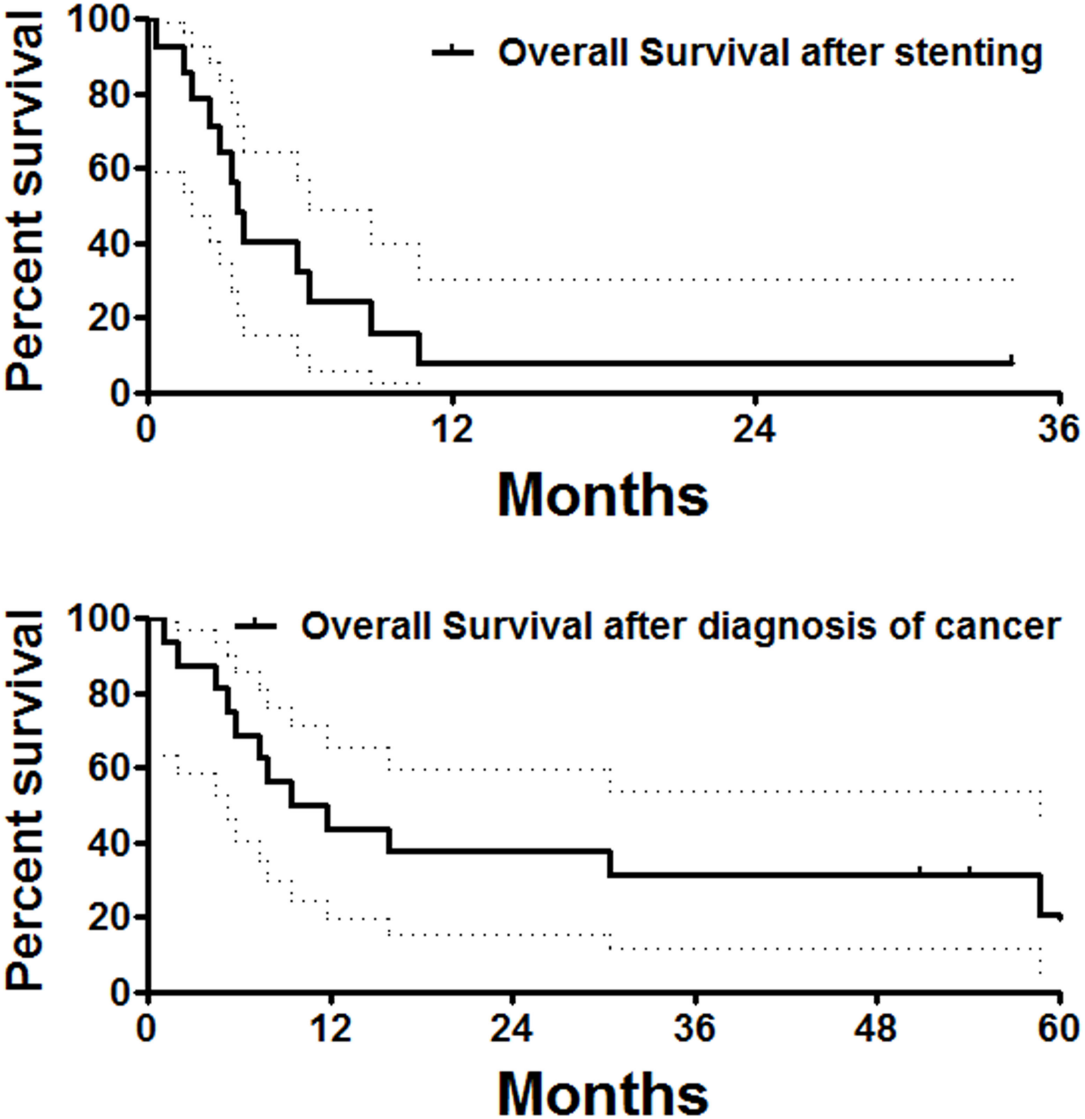
Overall survival follow-up. The median overall survival calculated from stent placement or diagnosis of the esophageal cancer was 3.5 and 10.6 months, respectively.

Except for two perioperative deaths and one patient lost to follow-up, all remaining 14 patients were followed up within a median period of 3.4 months after stent placement. At the end of follow-up, two patients had survived without obvious symptoms, and a total of 12 patients were dead owing to tumor progression (*n* = 10), severe infection (*n* = 1), and cerebrovascular accident (*n* = 1). The median overall survival was 13.8 months (**Figure 4**). There was no significant difference in overall survival between male and female patients.

## Discussion

Esophageal hemorrhage is a big challenge for patients with advanced esophageal cancer, and occurs in approximately 1%–5% of this patient group (5). The esophagus is a hollow organ, meaning that during bleeding, blood flows from the esophagus into the stomach, where it is difficult for compression or spontaneous hemostasis to occur. Thus, esophageal hemorrhage is most likely to be fatal. Endoscopic treatment modalities are first-line treatments of tumor hemostasis (6). However, rebreeding occurs frequently despite the variety of endoscopic interventions (7).

The insertion of SEMSs is usually used in the palliative treatment of malignant esophageal stricture in patients with recurrent or advanced esophageal cancer (8–10). Fully covered SEMSs have been used as a means of alternative management for esophageal perforation and fistula (19–21), as well as a salvage therapy for patients with esophageal variceal bleeding caused by cirrhosis (11, 12, 22, 23).

It is hard to find any studies about stenting treatments of fully covered SEMSs for malignant gastrointestinal hemorrhage, although there are some positive case reports about the use of stenting for refractory hemorrhage. For example, it has been reported that the placement of a fully or partially covered SEMS was effective for an intractable malignant hemorrhage caused by unresectable duodenal cancer (13, 14, 24). Yen et al. (14) also reported that, in a patient with duodenal cancer and multiple liver metastases, hematochezia stopped soon after the insertion of a partially covered SEMS (inserted by applying compression pressure on the cancer surface). Relatedly, D’Souza et al. (24) successfully induced hemostasis with the insertion of fully covered SEMS in a patient with uncontrollable duodenal hemorrhage caused by invasion of local recurrent hepatocellular carcinoma.

Currently, only a few case reports or a small number of case series report the use of fully covered SEMSs for esophageal cancer to achieve hemostasis after the failure of conventional endoscopic interventions to do so (6, 12, 15). Zhou et al. described a successful hemostasis with the placement of a fully covered SEMS in four patients with malignant esophageal hemorrhage (12). Bilal et al. (6) inserted a fully covered SEMS in a patient with a diffusely bleeding esophageal tumor with a severe luminal obstruction, which resulted in complete hemostasis and the resolution of symptoms. Isono et al. (15) also successfully induced hemostasis in a patient with a hemorrhage caused by esophageal tumor after esophageal stent placement.

In addition, massive esophageal hemorrhage is also observed following SEMS insertion in patients with malignant esophageal stricture or fistula (16, 17). Prior history of radiotherapy, the presence of esophageal fistula, and concomitant airway stent insertion are major factors contributing to esophageal hemorrhage after stent insertion for patients with esophageal cancer (17, 18). Thus, the role of fully covered SEMSs in the management of hemorrhage caused by esophageal cancer has not been established.

Our present study demonstrates that the insertion of a fully covered SEMS can be a salvage therapy tool for the management of esophageal cancer-related hemorrhage. SEMS insertion was technically successful in all patients, with no procedure-related deaths, and the compression hemostasis success rate was 88.2%. Although the insertion and removal of fully covered SEMS is not free from complications, our study showed a low rate of complications, that is major complications were observed in only 29.4% of patients. Stent migration was observed in only two patients, with a migration rate of 11.8%, which was lower than previous reports. Stent restenosis was also observed in two patients. Stent removal was performed in only two patients because of complications (25, 26). In our study, and in a few others, the placement of fully covered SEMSs could be both a safe and effective means of the salvage management of refractory malignant esophageal hemorrhage.

In addition, previous systemic treatments are most likely an important cause of esophageal bleeding. Only therapy refractory bleeding cases should be managed by the insertion of fully covered SEMS, considering that endoscopic treatment modalities are first-line treatments (although the technical success rate was 100% for all patients with stent placement indications, and the stent placement process did not induce or aggravate hematemesis in this study). Furthermore, there are contraindications to the insertion of esophageal stents. For example, esophageal stent placement may cause airway compression and asphyxia in patients with malignant airway stricture without airway stent placement. Stent intolerance, or even the development of laryngeal edema after stent placement in patients with proximal esophageal cancer, are additional complications associated with this procedure.

There were some limitations in our study. This is a retrospective study performed in a single center, over a long period of time (2019–2022), and on only 17 cases. Statistical significance and a comparison study is not expected for subgroups analysis in this pilot study because of its small sample size. Different types of SEMSs were used, including the segmental radioactive metal stent, the Bonastent and the Ultraflex stent, which may confer bias. In addition, transarterial embolization and chemoembolization procedures were not performed on all patients. Large studies and more data are required for statistical validation.

## Conclusion

Insertion of fully covered SEMSs is a safe and effective means of the salvage management of refractory esophageal cancer-related hemorrhage, and may be a way forward toward its innovative use of compression hemostasis.

## Data availability statement

The raw data supporting the conclusions of this article will be made available by the authors, without undue reservation.

## Ethics statement

Ethics approval was not provided for this study on human participants because ethics approval was waived by the Institutional Review Board of University due to its retrospective nature. The patients/participants provided their written informed consent to participate in this study.

## Author contributions

Conception and design: all authors. Data acquisition: all authors. Data analysis: YB. Data interpretation: all authors. Drafting the manuscript: YB. Critical revision of the manuscript: all authors. Statistical analysis: YB. Supervision: XH. All authors contributed to the article and approved the submitted version.
